# Placebo, Nocebo, and Machine Learning: How Generative AI Could Shape Patient Perception in Mental Health Care

**DOI:** 10.2196/78663

**Published:** 2025-08-15

**Authors:** Charlotte Blease

**Affiliations:** 1 Department of Women's and Children's Health, Faculty of Medicine, Uppsala University, MTC‑huset (MTC Building), Dag Hammarskjölds väg 14B, 1 tr, Uppsala, 75237, Sweden, 46 18 471 00 00

**Keywords:** ChatGPT, generative language models, large language models, placebo effects, nocebo effects, ethics, artificial intelligence, health disparities

## Abstract

The emergence of generative artificial intelligence (GenAI) in clinical settings—particularly in health documentation and communication—presents a largely unexplored but potentially transformative force in shaping placebo and nocebo effects. These psychosocial phenomena are especially potent in mental health care, where outcomes are closely tied to patients’ expectations, perceived provider competence, and empathy. Drawing on conceptual understanding of placebo and nocebo effects and the latest research, this Viewpoint argues that GenAI may amplify these effects, both positive and negative. Through tone, assurance, and even the rapidity of responses, GenAI-generated text—either co-written with clinicians or peers, or fully automated—could influence patient perceptions in ways that mental health clinicians may not currently fully anticipate. When embedded in clinician notes or patient-facing summaries, AI language may strengthen expectancies that underlie placebo effects, or conversely, heighten nocebo effects through subtle cues, inaccuracies, or potentially via loss of human nuance. This article explores the implications of AI-mediated clinical communication particularly in mental health care, emphasizing the importance of transparency, ethical oversight, and psychosocial awareness as these technologies evolve.

## Introduction

Unlike traditional search engines that return lists of web links, the new wave of chatbots—such as OpenAI’s GPT, Google’s Bard, and Bing AI—responds to user queries with conversational, human-like text. Powered by large language models (LLMs), these systems draw on vast datasets and probabilistic algorithms to generate fluent, contextually relevant responses. Their ability to recognize, summarize, and produce complex content has driven rapid adoption across sectors, including health care. This surge in use has sparked urgent debates about the safety, accuracy, and efficiency of generative artificial intelligence (GenAI) in clinical settings [1]. Despite the accelerated use of GenAI tools in health care, so far, little attention has been paid to the subtle psychological mechanisms that may be engaged by AI-generated communication, especially in mental health settings. Indeed, mental health care has always been a space where language not only influences treatment but, in the case of psychotherapy, can constitute it [2].

This Viewpoint article offers a conceptual exploration of one psychosocial aspect of care: how GenAI could strengthen placebo or nocebo effects. These psychological phenomena, which can exert both positive or negative effects on our health outcomes, are deeply rooted in patient expectations, perceptions of clinician competence and empathy, and relational trust in the clinic [3]. I propose that GenAI may enhance both placebo and nocebo effects. I also explore the ethical implications of this novel pathway in mental health care, exploring why GenAI has the potential to shift the foci of longstanding debates on practice dilemmas surrounding placebo and nocebo effects.

## How AI Could Influence Placebo and Nocebo Effects

Placebo and nocebo effects have traditionally been thought to arise in brick-and-mortar clinic environments. These effects are shaped by what clinicians say and do, that is, how they present information, express confidence, or deliver treatments [4]. However, patients’ own expectations—shaped by prior experiences, conditioning, and social learning—can also play a key role in their responses [5,6] In the age of digital tools, placebo and nocebo effects may be increasingly shaped through virtual, asynchronous channels [7-9].

For example, previous research suggests that patients often interpret online clinical notes more deeply than clinicians expect [10]. Patient-accessible records, though remote and asynchronous, may serve as a channel through which placebo and nocebo effects are shaped [8,11]. A clearly worded, hopeful, and empathetic summary could enhance placebo effects by shaping more positive expectations about recovery and therapeutic alliance. In contrast, clinical communications that subtly convey hopelessness or diagnostic pessimism, or which contain significant errors may contribute to loss of trust and anxiety, in turn influencing nocebo effects. Relatedly, in a variety of digital tools, “digital placebo effects” are thought to arise from features of online or app-based health interventions, such as interface design or ease of use, that shape patient expectations with respect to interventions [7,12].

It is important to emphasize that direct empirical evidence is still lacking, and much more research focus is needed to examine placebo and nocebo effects in digital contexts. Nonetheless, there are plausible reasons to believe that GenAI tools might elicit these effects. This shift is partly driven by the tendency of GenAI tools to evoke anthropomorphism among users [13,14], that is, people often, unintentionally, lapse into perceiving these systems as human-like agents, attributing to them qualities such as empathy, intent, and competence. These human-like qualities can, in turn, shape patient expectations and emotional reactions—key drivers of placebo and nocebo effects—by fostering a sense of optimistic reassurance or, conversely, anxiety.

First, consider placebo effects. One of the most consistent features of GenAI tools is the speed, fluency, and confidence with which it generates text. Whether summarizing a clinical encounter, or drafting follow-up instructions, studies suggest that GenAI has the potential to appear more coherent and polished than time-pressured human clinicians [15]. This fluency may signal competence to patients, perhaps more so than hastily typed or fragmented notes, replete with typos and acronyms, written by overburdened clinicians. The result may be that such tools thereby strengthen placebo effects. For example, studies show that clinical notes generated with GenAI tools—including “listening AI” that transcribes visits directly into electronic health records—are typically longer, and may do a better job of unpacking and explaining medical terms, than those written solely by clinicians [16-18].

Signals of empathy conveyed by GenAI tools may also augment placebo effects. A growing body of research shows that GenAI often conveys rich cues of empathy, particularly cognitive empathy, reflected in its ability to identify patients’ mental states, and compassion*,* reflected in signature expressions of concern and pro-social engagement [17,19,20]. Notably, as researchers emphasize, these empathetic signals contrast sharply with the often detached tone of physician-authored clinical documentation [17,20]. For example, GenAI tools have demonstrated a surprising ability to interpret emotional states, and in some cases, even outperform clinicians on measures of social intelligence [21,22]. A recent study tested whether people could distinguish between therapist- and AI-generated responses. Surprisingly, ChatGPT-4.0 not only proved difficult to distinguish from human therapists but was also rated higher on core therapeutic principles [19]. In one blinded experiment, physicians rated ChatGPT as 10 times more empathetic in written responses to patients’ queries on an online social media platform [17].

On the flip side, GenAI could also amplify nocebo effects by augmenting negative patient expectations. Tools such as ChatGPT are renowned for making authoritative sounding errors (known as “hallucinations”), and a high proportion of recommendations elicited by these tools can be egregiously wrong in health contexts [23]. From a patient perspective, such errors—if detected, or even if feared—might trigger increased anxiety or worry.

Similarly, when AI adoption erodes trust or undermines confidence in providers, it may heighten patient anxiety, amplifying nocebo effects and increasing the risk of adverse health outcomes. For example, some may find the responses offered by AI, which are designed to be agreeable and user-friendly, instead to be obsequious or lacking in authenticity [20,24]. It is conceivable that for these individuals, GenAI chatbots may prompt cynicism, either leading to disengagement, or even nocebo effects. According to the latest survey research, public skepticism toward AI in health care remains high. A 2023 study in *JAMA Network Open* found that nearly two-thirds of American adults express distrust that health care systems will use AI responsibly, with women showing even greater concern [25]. Crucially, this distrust was not linked to health literacy or AI knowledge. A year later, the 2024 KFF health tracking survey echoed these concerns: 56% doubted the accuracy of chatbot-generated health advice, and most remained uncertain about AI’s role in accessing reliable health information; only 21% saw it as helpful, while 23% believed it does more harm, and 55% were unsure [26].

## GenAI: A New Clinical Communicator

A growing body of survey research shows that clinicians, including psychiatrists, are incorporating GenAI tools into clinical practice, with the fastest task adoption for documentation [27-29]. Patients are also increasingly turning to these tools to seek health information or for socioemotional support [30]. For example, in 2024, a KFF health tracking survey found that GenAI chatbots like ChatGPT emerged as a popular source of health information, with 17% of American adults using them monthly, rising to 25% among those aged under 30 years [26]. Emerging studies show that patients derive benefits from using these tools to disclose and discuss socially sensitive or emotional concerns, offering round-the-clock support with perceived comfort of anonymity and lack of human judgment [30].

The adoption of GenAI tools as a new communicative “agent” in health care may meaningfully shape placebo and nocebo effects, long-recognized phenomena in clinical research. As noted, the placebo effect is a genuine biopsychosocial phenomenon wherein patient expectations, influenced by factors like provider communication, treatment rationale, and the care environment [3,4,31], can lead to meaningful symptom alleviation, particularly for pain, depression, and anxiety [3,32]. Conversely, nocebo effects involve negative expectations that lead to adverse outcomes, triggered by concerns about side effects, risks, or aspects of treatment delivery or design [3,11]. Some researchers propose that psychotherapy may also be a setting where nocebo effects emerge, particularly when clinicians unintentionally convey subtle negative suggestions about treatments, symptoms, or prognoses, or when the therapeutic alliance is weakened by a lack of trust [33].

## Old Ethics, New Tech: Placebo, Nocebo, and Practice Dilemmas

Long-standing, substantial academic literature has focused on the ethics of placebo and nocebo effects. This body of literature has focused almost entirely on how clinicians, primarily physicians [34], communicate with patients, examining both verbal and nonverbal cues with only limited attention given to how clinical artifacts and environmental features may also influence placebo and nocebo effects [35]. The result is that almost exclusively, the focus of ethical attention in placebo studies, including in mental health care, has hitherto been on provider communication. As GenAI tools become increasingly enmeshed in clinical care, especially in mental health settings, their potential to benefit patients, but also cause harm, must not be ignored [1,36].

To fully evaluate the ethicality of these tools, they cannot be considered in isolation; it is also imperative to explore how these tools compare to traditional human-mediated care, including whether they may fare better or worse for patients along a variety of ethical concerns [36]. Below, I offer exploratory—rather than exhaustive—reflections on how the advent of GenAI in health care may reframe longstanding ethical dilemmas surrounding placebo and nocebo effects.

Consider the most well-known ethical dilemma pertaining to placebo studies: the administration of placebos in clinical contexts. Here, placebos may be offered to patients with the intention of harnessing beneficial effects, even while doing so deceives the patient. Most medical ethicists strongly oppose this deception, emphasizing that it undermines trust, compromises patient autonomy, and promotes a paternalistic model of care [37].

With GenAI, the deception operates at a different level. It’s not just about a physician misleading a patient about a pill; it may involve patients being misled about who they are interacting with. Placebo effects may be amplified through this illusion, but if patients believe they’re communicating with a human when it’s actually a chatbot, trust is at risk. A relevant parallel can be drawn from the well-known “open–hidden” paradigm in placebo research, where patients receiving identical treatments experience significantly stronger effects when the intervention is administered openly by a clinician rather than covertly [38,39]. These findings underscore how the source and visibility of a therapeutic act, not just its content, can powerfully shape outcomes. Similarly, patients’ perceptions of whether they are interacting with a human or a chatbot may critically influence both placebo and nocebo effects. Although not directly indicative of placebo or nocebo effects, in one notable case, the company Koko, which provides online mental health support, issued a public apology in January 2023 after using ChatGPT to generate emotional responses while misleading users into believing they were written by real people [40].

Beyond issues of identity, LLMs themselves can be sources of deception [23]. Some models have been shown to produce false or misleading responses, and in certain contexts, even reportedly admitting to doing so [41]. This raises further ethical concerns about trust, transparency, and oversight in patient-facing AI tools.

Complicating matters, even when patients are aware, or later discover, that they were interacting with AI, placebo effects may be dampened but not entirely lost, with meaningful psychological or therapeutic benefits still emerging. For example, a study found that AI-generated messages made recipients feel more understood than human-written ones, and AI was better at detecting emotions [42]. Yet, once recipients learned the messages came from AI, this effect diminished. However, when comparing transparently labeled AI and human responses, participants rated them almost identically. Notably, however, this does not resolve the ethical tension between respecting patient autonomy and pursuing beneficence through placebo mechanisms. Nevertheless, the study does highlight the complexity of perception, expectation, and ethical transparency in AI-mediated care, suggesting that patients may yet derive placebo effects even when they know they’re “talking to” AI.

Another well-recognized ethical challenge lies in balancing the duty of honesty—such as disclosing potential side effects of medications—with the risk of inducing harm through nocebo effects, where negative expectations may contribute to self-fulfilling adverse outcomes [43]. Here, it has been proposed, that one way to resolve the ethical dilemma is to reframe information in ways that are truthful but which influence more positive outcomes [44].

For instance, rather than stating about a treatment or intervention, “side effects of this drug are quite common—20% of people experience them,” it may be more effective—and equally truthful -to say, “80% of people do not experience side effects.” GenAI tools, with their capacity to rapidly adjust language and tone, offer a promising means of delivering such positively reframed information. Unlike human clinicians, whose communication may be affected by burnout, variability, or limited time, GenAI could help consistently implement ethically sensitive messaging, offering a potential solution to this longstanding ethical dilemma.

In addition, consider a more overshadowed ethical issue in placebo studies: justice [45,46]. This concern lies in the unequal distribution of harm that may arise from differences in patient–clinician communication. Research shows that patients from marginalized groups—such as those who are racially minoritized, have lower incomes, higher body weight, or psychiatric diagnoses—often receive lower-quality communication, less empathy, and more negative framing in clinical encounters [46]. These disparities reduce placebo effects, and heighten negative expectations, increasing susceptibility to nocebo effects. Thus, nocebo-related harm may disproportionately affect already disadvantaged populations by compounding existing health inequities, therby raising serious concerns about fairness and justice in care.

GenAI may help bridge some face-to-face health communication disparities by offering more consistent, high-quality interactions that are not influenced by appearance [47]. Unlike face-to-face encounters, GenAI chatbots do not discriminate based on race, body size, or other visible traits. They also provide scalable access to health information, allowing users to engage at their own pace and literacy level, with customizable tone and style. Notably, the 2024 KFF Health Tracking Survey found that Black (23%) and Hispanic (31%) adults were more likely than White adults (16%) to say AI chatbots help them find accurate health information [26]. Similarly, 51% of Black and 44% of Hispanic adults reported trusting AI-provided advice, compared to just 29% of White adults, highlighting how GenAI may be perceived as a tool for expanding access in historically underserved communities.

However, the promise of GenAI to reduce disparities, including nocebo-related harms, remains contingent on addressing persistent digital divides [48]. Patients most vulnerable to health inequities may also face barriers to accessing the very tools designed to support them, such as the lack of digital devices, broadband, or sufficient digital literacy to effectively use GenAI chatbots [48]. Without tackling these structural barriers, GenAI risks reinforcing, rather than resolving, existing inequities.

## Conclusion

As GenAI begins to generate the words patients read—whether within patient accessible records, online messaging with peer support or clinicians, or via fully automated chatbot dialogs—it may influence psychosocial aspects of care. While the concerns of this Viewpoint are exploratory in nature, the arguments rest on emerging research, and further empirical studies are needed to investigate how GenAI may shape placebo and nocebo effects when it comes to real-world mental health care. Understanding how these tools shape patient perceptions is not a peripheral concern; it is central to the future of ethical, effective mental health care. Future research should include qualitative and co-design approaches to center patient voices and explore how patients with mental health conditions perceive, interpret, and respond to GenAI in clinical contexts [49]. Indeed, while this Viewpoint has focused on mental health, similar mechanisms are likely at play across many areas of medicine, and future work should explore how generative AI may amplify or mitigate placebo and nocebo effects in other clinical contexts, including chronic illness and polypharmacy. Not only what is written, but how it is phrased, may influence patient behavior and outcomes, via placebo and nocebo effects. It is time for the fields of placebo studies and health communication to enter a new era of research.

## Abbreviations

GenAI: generative artificial intelligence
LLM: large language model
